# The status of dermatoglyphics as a biomarker of Tel Hashomer camptodactyly syndrome: a review of the literature

**DOI:** 10.1186/s13256-016-1048-7

**Published:** 2016-09-20

**Authors:** Buddhika T. B. Wijerathne, Robert J. Meier, Suneth B. Agampodi

**Affiliations:** 1 Department of Community Medicine, Faculty of Medicine and Allied Sciences, Rajarata University of Sri Lanka, Saliyapura, 50008 Anuradhapura Sri Lanka; 2 Department of Anthropology, Indiana University, Bloomington, IN 47405 USA

**Keywords:** Tel Hashomer camptodactyly syndrome, Dermatoglyphics, Camptodactyly

## Abstract

**Introduction:**

Tel Hashomer camptodactyly syndrome is a rare disease and only a few cases have been reported. Dermatoglyphics potentially provide relevant phenotypic biomarkers that were initially noted as a vital clinical feature of this disease. Dermatoglyphics possibly can indicate growth disturbances that took place during early fetal development at the time when epidermal ridges were being formed into discernable patterns. Consequently, these intrauterine effects might well have occurred in association with the expression of the Tel Hashomer camptodactyly syndrome. Therefore, this review was undertaken to provide, as far as we know, the first attempt to broadly assess dermatoglyphic features that are connected with the Tel Hashomer camptodactyly syndrome. If a developmental association between dermatoglyphics and Tel Hashomer camptodactyly can be firmly established, this would probably document that Tel Hashomer camptodactyly disease has its origins during the early fetal period.

**Methods:**

A systematic literature search was conducted using articles from PubMed (Medline), POPLINE, Trip Database, Cochrane Library, and gray literature up to 31 March 2015. The review was performed according to the Preferred Reporting Items for Systematic Reviews and Meta-Analyses statement.

**Results:**

Fourteen relevant publications were included in the review. There were 23 cases of patients with Tel Hashomer camptodactyly syndrome that were described in these published articles. We reviewed the dermatoglyphics of 21 available cases out of all of the published and electronically available cases of Tel Hashomer camptodactyly. Eight cases reported whorls to be the most common digital pattern with an expected rise of ridge count. Two cases show significantly high frequencies of arch patterns. Further, there were increased numbers of palmar creases, along with abnormal flexion creases or other palmar dermatoglyphic abnormalities reported in all cases.

**Conclusion:**

This review highlighted the desirability of thoroughly observing and recording dermatoglyphic features when reporting on future patients with Tel Hashomer camptodactyly syndrome, in conjunction with carrying out modern molecular methods.

## Introduction

Tel Hashomer camptodactyly (THC) syndrome is a rare disease first termed by Goodman *et al*. in 1976 after examining two sisters with camptodactyly [1]. Earlier in 1972, they reported two brother and sister pairs having similar clinical features [2]. Up to the present time, a literature search has found only 23 cases. THC is mainly characterized by the presence of camptodactyly with muscular hypoplasia and weakness, skeletal dysplasia, facial dysmorphism (facial asymmetry, small mouth, broad nasal bridge, long philtrum, and hypertelorism), and abnormal dermatoglyphics: Online Mendelian Inheritance in Man® (OMIM) #211960, The portal for rare diseases and orphan drugs (ORPHA) 3292 [3, 4]. In addition, mitral valve prolapse, spina bifida, scoliosis, inguinal hernia, winging scapulae, clubbed feet, syndactyly and clinodactyly were indicated as clinical features [3, 4]. THC is considered to be a disease with autosomal recessive inheritance [5]. Mochizuki *et al*. [6] recently reviewed the molecular characteristics of a patient described by Toriello *et al.* in 1990 [7] and suggested that at least several cases of THC may actually be Ehlers–Danlos syndrome.

Goodman *et al.* [1] stated the importance of dermatoglyphic biomarkers as clinical features when diagnosing THC. Dermatoglyphic characters that need to be present to diagnose THC are: (a) presence of seven or more whorls on digits (these whorls extend beyond the borders of the terminal phalanges), (b) low main line index, caused by the highly vertical orientation of the A to D radiants, and (c) numerous palmar creases that obliterate the normal structure of the ridges and openings of the sweat pores. We systematically analyzed all published cases of THC syndrome to describe the importance of dermatoglyphics in diagnosing this rare disease.

## Methods

The review has been conducted and reported using the Preferred Reporting Items for Systematic Reviews and Meta-Analyses (PRISMA) statement guidelines [8].

### Search strategy

We conducted a search of the literature for articles indexed in PubMed® (Medline), POPLINE, TRIP Database, and Cochrane Library database, from earliest dates to 31 March 2015. In addition, we searched the gray literature sources of Google Scholar, OpenGrey, and Google, from earliest date to 31 March 2015. The reference lists of the studies selected were manually searched for any relevant studies. We did not restrict the searches based on language or publication status. The following terms were used to search the literature: “Tel Hashomer camptodactyly”, “Tel Hashomer camptodactyly syndrome.”

### Eligibility criteria and data extraction

All studies that had diagnosed and reported THC syndrome were selected. From each, the following details were extracted: disease diagnosis, demographic details (age, sex, consanguinity, ancestry/lineage, country of case reported), and dermatoglyphic features. Initially, the full texts and abstract were screened and extracted by BTBW, and later SBA and RJM independently reviewed these studies for accuracy.

## Results

**Fig. 1:**
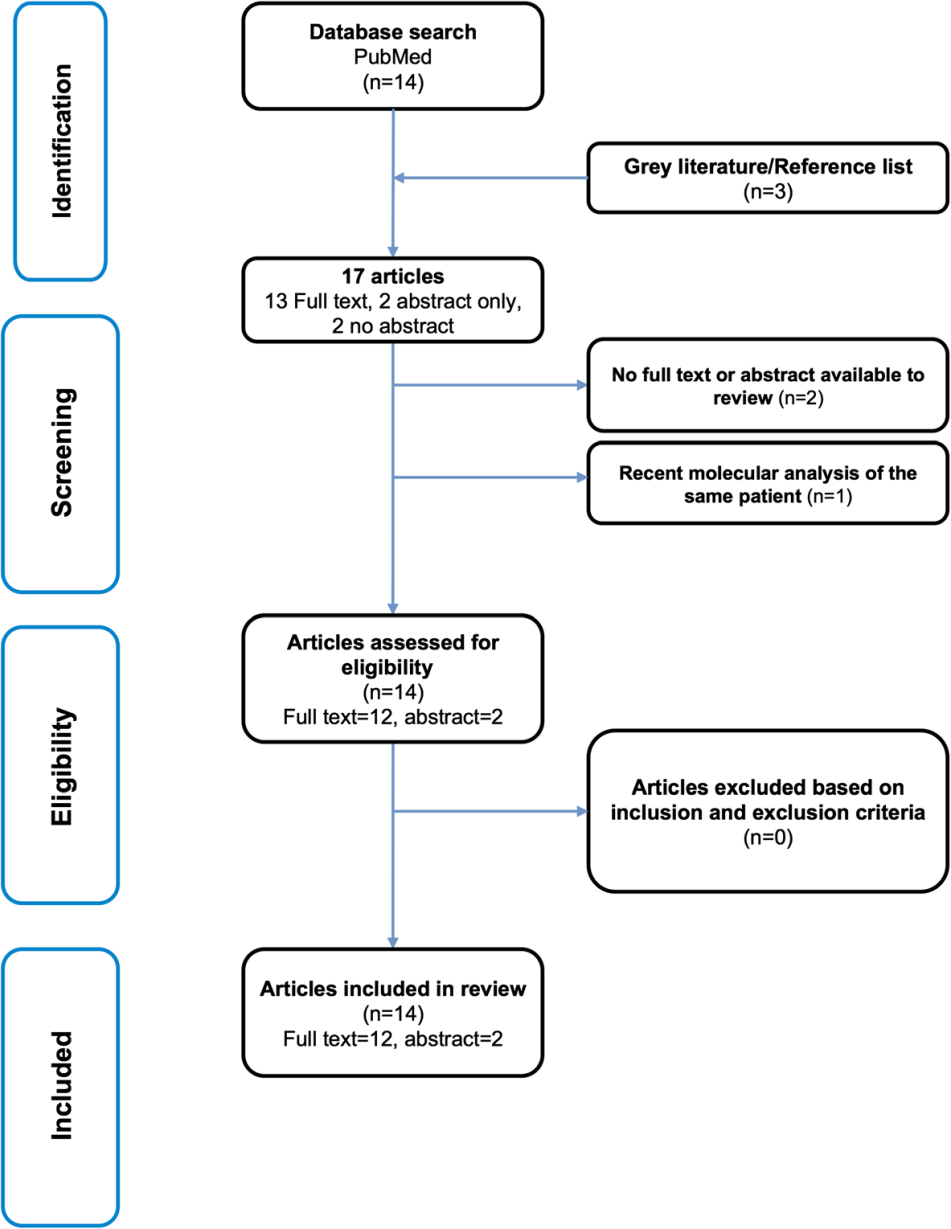
A flow diagram depicting the review process and study selection

The search of electronic databases yielded 14 publications. In addition, three publications were obtained from gray literature sources and hand searching the reference lists (Fig. 1). Full texts are available for 13 publications [1, 2, 5–7, 9–16], only an abstract was available for two publications [17, 18], and an abstract or full text was unavailable for another two [19, 20]. Out of all 17 studies, only 14 publications were reviewed due to the unavailability of records for two publications [19, 20] and one publication reanalyzed a patient whose dermatoglyphics had been described previously [6].

There were 23 cases of THC described in the reviewed publications [6]. Six cases reported from Israel [1, 2, 5], three from Brazil [9, 12], three from India [14, 16], two from Italy [13, 18], three from Poland [10], one from the UK [11], two from the USA [7], two from Russia [17], and one from Hungary [15]. There were 11 females and nine males, and for three patients their sex was not reported in the available abstract [17, 18]. All reported cases were among siblings or first-degree relatives. Eleven cases were born to consanguineous parents [1, 9, 10, 13, 16] while nine were born to non- consanguineous parents [2, 5, 11, 12, 14, 15]. Two cases did not report the consanguinity of their parents [7] and in three cases the consanguinity was not reported in the available abstract [17, 18]. The dermatoglyphics were not reported in one case [16] and for another case dermatoglyphics were not reported in the abstract [18].

Key findings on dermatoglyphic features of the cases of THC are summarized in Table 1.

**Table 1:** Dermatoglyphic features of published cases of Tel Hashomer camptodactyly syndrome

Author and year	Disease diagnosis	Demography (age, sex, consanguinity, ancestry/lineage, country of case reported)	Dermatoglyphic features
Goodman *et al*., 1972 [2]	Camptodactyly with muscular hypoplasia, skeletal dysplasia, and abnormal palmar creases Clinically diagnosed	17-years old Female (Proposita) No consanguinity Jewish Moroccan ancestry Reported from Israel	Digital dermatoglyphics: Whorls = 8 and extended beyond the borders of the terminal phalanges, TRC high = 271 Palmar dermatoglyphics: A–b ridge count = 73, ridge breath = 606, maximal atd angle = 110.5, modification of creases
		13-years old Male (affected brother) No consanguinity Jewish Moroccan ancestry Reported from Israel	Digital dermatoglyphics: Whorls=8 and extended beyond the borders of the terminal phalanges, TRC high = 350 Palmar dermatoglyphics: A–b ridge count=120, ridge breath=536, maximal atd angle=101.0, modification of crease
Goodman *et al*., 1976 [1]	Tel Hashomer camptodactyly syndrome Clinically diagnosed	20-years old Female (Proposita) Consanguineous parents Bedouin Reported from Israel	Digital dermatoglyphics: Whorls = 9, TRC high = 177 Palmar dermatoglyphics: A–b ridge count = 77, ridge breath = 493, maximal atd angle = 75, modification of crease
		19-years old Male (affected brother) Consanguineous parents Bedouin Reported from Israel	Digital dermatoglyphics: Whorls = 7, TRC high = 209 Palmar dermatoglyphics: A–b ridge count = 76, ridge breath = 697 maximal atd angle = 92, modification of crease
Gollop and Colletto 1984 [9]	Tel Hashomer camptodactyly syndrome Clinically diagnosed Normal female chromosome	7-years old Female (Proposita) Consanguineous parents Brazilian Reported from Brazil	Digital dermatoglyphics: Arches = 6, whorl = 2, ulnar loop = 2 TRC (LH = 11, RH = 14) Palmar dermatoglyphics: Bilateral transverse palmar crease, numerous additional palmar creases, vertical orientation of lines a and t, left hand the ulnarity index of patient 1 was decreased (0.51; mean Brazilian normal value 0.79 and 0.08, a–b RC (LH = 33, RH = 32), a–d RC (LH = 44, RH = 45), atd angle (LH = 40, RH = 35), main line index = (LH = 7, RH = 7)
		9-years old Male (affected brother) Consanguineous parents Brazilian Reported from Brazil	Digital dermatoglyphics: Arches=9 UL 1, TRC (LH = 0 RH = 1) Palmar dermatoglyphics: Bilateral transverse palmar creases, numerous white lines, and vertical orientation of lines a and t, the b triradius is absent and the atd angle is increased owing to a rare ulnar loop present in the hypothenar region at the level of the transverse palmar crease A–b RC = (LH = 0, RH = 42), a–b RC = (LH = 64, RH = 66), atd angle (LH = 107, RH = 106), main line index = (LH = 5, RH = 7)
Tylki-Szymanska 1986 [10]	Tel Hashomer camptodactyly syndrome Clinically diagnosed	13-years old Female (Proposita) Consanguineous parents (first cousins) Libyan family Reported from Poland	Abnormal hand prints +
		13-years old Male (Proposita) Consanguineous parents (first cousins) Libyan family Reported from Poland	Abnormal hand prints +
		10-years old Female (Proposita) Consanguineous parents (first cousins) Libyan family Reported from Poland	Abnormal hand prints +
Patton *et al*., 1986 [11]	Tel Hashomer camptodactyly syndrome Clinically diagnosed	4-years old Female (Proposita) No consanguinity Mother was English and father was Anglo-Asian origin Reported from UK	Palmar dermatoglyphics: Absent or decreased interphalangeal creases
Pagnan *et al*., 1988 [12]	Tel Hashomer camptodactyly syndrome Clinically diagnosed Karyotype: chromosomes were normal (46,XX)	4½-years old Female (Proposita) No consanguinity Ancestry : NR Reported from Brazil	Digital dermatoglyphics: Whorls 10/10, digital patterns are large, with displacement of triradii, TRC = 350 Palmar dermatoglyphics: A–b RC = 95, maximal atd 108.5, a–d count = 97, MLI = 16, modification of crease, simian crease of left hand, many “white lines” (shallow grooves of different length, width, and direction) on both palms and fingers and vertical orientation of the a, b, and t lines on the right and left hand
Toriello *et al*., 1990 [7]	Tel Hashomer camptodactyly Clinically diagnosed	15½-years old Male (Proposita) Consanguinity: NR Hispanic Reported from USA	Digital dermatoglyphics: Large whorls on each digit Palmar dermatoglyphics: A–d triradii, vertical a, b, and t lines, multiple white lines, and 2 palmar whorls on each hand
		11-years old Female (younger sister) Consanguinity: NR Hispanic Reported from USA	Digital dermatoglyphics: 9 large whorls and 1 ulnar loop Palmar dermatoglyphics: A triradius, vertical a, b, and t lines, and multiple white lines
Franceschini *et al*., 1993 [13]	Tel Hashomer camptodactyly syndrome Clinically diagnosed	17-years old Female (Proposita) Consanguineous parents (first cousins) Ancestry: NR Reported from Italy	Palmar dermatoglyphics: Bilateral transverse palmar creases, numerous additional palmar creases (so-called “white lines”), and ulnar displacement of t triradius
Scarano *et al*. 1994 [18]	Tel Hashomer camptodactyly syndrome	Age: NRA Gender: NRA Consanguinity: NRA Ancestry: NRA Reported from Italy	Dermatoglyphics NRA
Rogovina *et al*., 1995 [17]	Tel Hashomer camptodactyly syndrome	Patient 1 (siblings) Age, sex and ancestry NRA Consanguinity: NRA Reported from Russia	Flexion folds between phalanges were absent Other dermatoglyphic features NRA
		Patient 2 (siblings) Age, sex and ancestry NRA Consanguinity: NRA Reported from Russia	Flexion folds between phalanges were absent Other dermatoglyphic features NRA
Patel and Adhia 2004 [14]	Tel Hashomer camptodactyly syndrome Clinically diagnosed Karyotype: 46,XX, chromosomal	30-years old Female (Proposita) No consanguinity Ancestry: NR Reported from India	Abnormal dermatoglyphics +
		Age: NR Female (sister) No consanguinity Ancestry: NR Reported from India	Abnormal dermatoglyphics +
Melegh *et al*., 2005 [15]	Tel Hashomer camptodactyly syndrome Clinically diagnosed Karyotype: normal	4-years old Male (Proposita) No consanguinity Hungarian, followed up for 12 years Reported from Hungary	Digital dermatoglyphics: Whorl patterns on all ten fingertips, no other unusual ridges or flexion creases Palmar dermatoglyphics: No other unusual ridges or flexion creases were seen on the fingers, palms, and soles
Smolkin *et al*., 2011 [5]	Tel Hashomer camptodactyly syndrome	Twin 1 monochorionic biamniotic 32-weeks 5-days old Male No consanguinity Ancestry: NR Reported from Israel	Digital dermatoglyphics: RH = absent interphalangeal crease in finger 5 LH = absent interphalangeal crease in finger 4, 5 Palmar dermatoglyphics: RH = simian crease LH = simian crease Partial absence of dermatoglyphic features
		Twin 1 monochorionic biamniotic 32-weeks 5-days old Male No consanguinity Ancestry: NR Reported from Israel	Digital dermatoglyphics: RH = absent interphalangeal crease in finger 2, 3, 4 LH = absent interphalangeal crease in finger 2, 3,4, 5 Palmar dermatoglyphics: RH = simian crease LH = simian crease Partial absence of dermatoglyphic features
Shah *et al*., 2013 [16]	Tel Hashomer camptodactyly syndrome	25-years old Male (Proposita) Consanguineous Ancestry: NR Reported from India	Dermatoglyphics not reported

*LH* left hand, *MLI* main line index, *NR* not reported, *NRA* not reported in abstract, *RC* Ridge count, *RH* right hand, *TRC* total ridge count, *UL* Ulnar loop

## Discussion

Of the 21 cases that could be evaluated, eight reported whorls to be the most common digital pattern [1, 2, 7, 12, 15]. Of particular interest, four of these were females with THC syndrome who had at least eight whorls [1, 2, 7, 12]. Conversely, normal males tend to have higher frequencies of whorl patterns [21]. As expected, there were also high average ridge counts, since whorls usually do have more ridges than loops and, of course, arches have zero ridge counts. It is also of interest to note that two of the cases, involving a sister/brother pair, had high frequencies of digital arch patterns with the brother having nine arches [9]. Usually, normal females tend to have more arches than males [21]. Furthermore, there frequently were an increased number of palmar creases than would normally be observed, along with abnormal flexion creases or other palmar dermatoglyphic abnormalities reported in all cases.

The fact that these cases appear to show some unusual results, for instance, in terms of digital patterns from unexpectedly high whorl frequency, especially in females with THC, to a very high number of arches, notably in males with THC, might indicate that there could have been some growth disturbances that took place during early fetal development at the time when epidermal ridges were being formed. In addition, unusual findings with respect to palmar dermatoglyphic features and flexion creases might well be indicative of abnormal developmental conditions. Of course, this intrauterine effect might well have occurred in association with the camptodactyly syndrome. Accordingly, it seems apparent that dermatoglyphic biomarkers may provide important clues when applying differential diagnoses, in conjunction with current molecular testing.

Therefore, it is important to thoroughly observe and record dermatoglyphic features when reporting future patients with THC syndrome, in addition to carrying out modern molecular methods. A highly beneficial consequence of this practice is that possible associations of dermatoglyphic biomarkers in genetically confirmed cases of THC could then be used as a relevant diagnostic aid in countries that have limited medical diagnostic resources.

## Abbreviation

THC: Tel Hashomer camptodactyly
